# Soluble Fc Receptor for IgM in Sera From Subsets of Patients With Chronic Lymphocytic Leukemia as Determined by a New Mouse Monoclonal Antibody

**DOI:** 10.3389/fimmu.2022.863895

**Published:** 2022-06-16

**Authors:** Pedram Mahmoudi Aliabadi, Ruth Teuber, Peter K. Jani, Landon Wilson, Philipp Enghard, Stephen Barnes, Nicholas Chiorazzi, Andreas Radbruch, Fritz Melchers, Hiromi Kubagawa

**Affiliations:** ^1^ Humoral Immune Regulation, Deutsches Rheuma-Forschungszentrum, Berlin, Germany; ^2^ Lymphocyte Development, Deutsches Rheuma-Forschungszentrum, Berlin, Germany; ^3^ Targeted Metabolomics and Proteomics Laboratory, Department of Pharmacology and Toxicology, University of Alabama at Birmingham, Birmingham, AL, United States; ^4^ Department of Nephrology and Medical Intensive Care, Charité-Universitätsmedizin, Berlin, Germany; ^5^ Karches Center for Oncology Research, Feinstein Institute for Medical Research, Manhasset, NY, United States; ^6^ Cell Biology, Deutsches Rheuma-Forschungszentrum, Berlin, Germany

**Keywords:** FcµR, soluble receptor, mAb, exosome, alternative splicing, CLL, autoimmunity

## Abstract

The FcR for IgM (FcµR) is the newest member of the FcR family, selectively expressed by lymphocytes, and distinct from FcRs for switched Ig isotypes that are expressed by various immune cell types and non-hematopoietic cells. From studies of *Fcmr*-ablated mice, FcµR was shown to have a regulatory function in B-cell tolerance, as evidenced by high serum titers of autoantibodies of the IgM and IgG isotypes in mutant mice. In our previous studies, both cell-surface and serum FcµR levels were elevated in patients with chronic lymphocytic leukemia (CLL), where antigen-independent self-ligation of BCR is a hallmark of the neoplastic B cells. This was assessed by sandwich ELISA using two different ectodomain-specific mAbs. To determine whether the serum FcµR is derived from cleavage of its cell-surface receptor (shedding) or its alternative splicing to skip the transmembrane exon resulting in a 70-aa unique hydrophilic C-terminus (soluble), we developed a new mouse IgG1κ mAb specific for human soluble FcμR (solFcμR) by taking advantages of the unique nature of transductant stably producing His-tagged solFcµR and of an *in vivo* differential immunization. His-tagged solFcμR attached to exosomes and plasma membranes, allowing immunization and initial hybridoma screening without purification of solFcμR. Differential immunization with tolerogen (membrane FcμR) and immunogen (solFcμR) also facilitated to generate solFcμR-specific hybridomas. The resultant solFcμR-specific mAb reacted with serum FcµR in subsets of CLL patients. This mAb, along with another ectodomain-specific mAb, will be used for verifying the hypothesis that the production of solFcµR is the consequence of chronic stimulation of BCR.

## Introduction

Receptors for the Fc portion of switched Ig molecules (*i.e.*, FcγRs, FcϵRs and FcαR) are expressed by a variety of immune cell types as well as by non-hematopoietic cells. Their interactions with IgG, IgE or IgA initiate a broad spectrum of effector functions, such as phagocytosis of Ab-coated microbes, Ab-dependent cell-mediated cytotoxicity, release of inflammatory mediators, and regulation of B cell responses. These diverse functions of FcRs depend upon their Ig ligands and the cell types expressing them. These FcRs are thus considered central mediators of Ab-triggered responses which couple the adaptive and innate immune responses (1). By contrast, FcR for evolutionally first appearing IgM molecules (FcµR), is the newest member of the FcR family and selectively expressed by lymphocytes: B, T and NK cells in humans and only B cells in mice, although there is disagreement about expression of FcµR by non-B cells in mice [see reviews (2, 3)]. Among five different *Fcmr-*ablated mouse strains, there are obvious discrepancies in the resultant phenotypes, but the common finding among these mutant mice is an impairment of B cell tolerance as evidenced by enhanced serum titers of autoantibodies of both IgM and IgG (2). *FCMR-*deficiency has not yet been identified in humans; however, if this defect occurred, the clinical abnormalities might be much more complex and profound than in *Fcmr-*ablated mice, because human FcµR is expressed by additional cell types (*i.e.*, T and NK cells). Thus, documentation of FcµR functions in humans relies on *ex vivo* experiments and there are several noteworthy findings. (*i*) Co-ligation of FcµR and other receptors (*e.g.*, Fas, BCR or CD2) on the same cell surface by agonistic IgM mAbs either inhibits Fas-mediated apoptosis or enhances BCR- or CD2-mediated Ca**^2+^** mobilization, suggesting a dual (negative and positive) signaling ability to cells (4–7). The *Cis* engagement dominates the *trans*, probably due to locally high concentrations of IgM ligands (6). (*ii*) FcµR is highly expressed by chronic lymphocytic leukemia (CLL) B cells (5, 8–11), and, after IgM binding, it is rapidly internalized in lysosomes *via* an endocytic pathway (11). FcµR is not only distributed on plasma membrane, but also accumulates in large pools in the *trans*-Golgi network (11). (*iii*) FcµRs on T and NK cells are dramatically down-modulated upon IL-2 stimulation *in vitro*, consistent with the findings that the surface levels of FcµR on freshly prepared effector memory T cells are much lower than naive T cells. Binding of IgM to FcµR on NK cells initiates intracellular signals but does not mediate NK cell cytotoxicity (7). (*iv*) FcµR-mediated IgM uptake by T cells enhances their surface expression of TCR and co-stimulatory molecules, thereby facilitating T cell activation particularly when Ag concentrations are low (12).

Regarding the relevance of FcµR in disease, there has been a strong association of FcµR with CLL for decades based on the findings that CLL B cells form rosettes with IgM Ab-coated erythrocytes or exhibit IgM binding by immunofluorescence [see review (2)]. We have also examined FcµR expression in CLL patients using receptor-specific mAbs, finding marked elevation of serum titers of FcµR in many CLL patients, but not healthy individuals (10), except for one person who developed high titers of anti-nuclear Abs two years later (2). Serum FcµR was resolved as an ~40 kDa protein, distinct from the ~60 kDa cell surface FcµR, and proteomic analysis revealed a soluble form of the receptor (solFcµR), created by alternative mRNA splicing that led to a skipping of the transmembrane exon, resulting in a unique 70-aa hydrophilic C-tail due to the reading frame shift (10). The assessment of serum FcµR was performed by sandwich ELISA using two different, FcµR ectodomain (EC)-specific mAbs. However, this sandwich ELISA did not distinguish the solFcµR of ~40 kDa from a cleaved form (shedding) of the membrane bound FcµR (≤~35 kDa), if it did exist.

In the present studies, we have focused on the development of mouse mAbs specifically recognizing the unique C-terminal portion of human solFcµR by taking advantages of the unique characteristics of its histidine (His)-tagged, stable transductant and of an *in vivo* differential immunization strategy without purifying solFcµR.

## Materials and Methods

### Transductants Stably Expressing SolFcµR

To develop cell lines stably producing recombinant solFcµR proteins, two different solFcµR constructs were synthesized by Eurofins Genetics (Berlin, Germany). *First*, the coding sequence of FcµR splice variant cDNA, which was originally identified in PMA-activated human pre-B cell line 697 (ADK11426 in NCBI) (10), was flanked by the restriction enzyme sites of *Bgl*II and *Cla*I site at the 5’ and 3’ sites, respectively (designated as native solFcµR). *Second*, we introduced a Kozak sequence “ACC” between the *Bgl*II site and the translation initiation site ATG and 18 nt (CATCACCATCACCATCAC) coding six His tag sequence before the translation termination site TGA and *Cla*I site into the above coding sequence (designated as solFcµR-His). After verifying the correct sequences, both constructs were subcloned into a bicistronic retroviral expression vector pRetroX-sGreen (Takara), transfected into PLAT-E and PLAT-A packaging cell lines, and respectively transduced into mouse plasmacytoma (Ag8.653) and human embryonic kidney cell (HEK) lines as previously described (5). After enriching GFP-positive cells by FACS, stable solFcµR transductants were thus established. In addition to these solFcµR transductants, several other stable transductants were also included as controls in the present study. These included: (*i*) Ag8 and BW5147 thymoma transductants stably expressing membrane form of human FcµR (Ag8 or BW memFcµR) and (*ii*) Ag8 transductant expressing His-tagged FcµR EC only (FcµR EC-His), along with GFP.

### ELISA for SolFcµR

To assess the solFcµR titers in culture supernatants of transductants and in serum samples, the sandwich (or indirect) ELISA was conducted as previously described (10). In brief, 96-well hard plates (Sarstedt, Nümbrecht, Germany) for culture supernatants and 96-well flexible plates (Costar) for serum samples were coated overnight at 4°C with 100 µl of purified mouse mAbs specific for FcµR EC [clones HM6, HM7 (both γ2bκ) and HM10 (γ1κ)] or solFcµR [clone HMD22 (γ1κ)] at the protein concentration of 10 µg/ml in borate saline buffer pH 8.3 (BSB) and masked with 300 µl of 1% BSA in BSB at room temperature for 1 hr. [Protein concentration was determined by Nanodrop spectrophotometer using an extinction coefficient of 1.4 as 1 mg/ml.] After washing with PBS or PBS/0.05% Tween 20, wells were incubated with 100 µl of serial dilutions of culture supernatants at 37°C for 2 hs or for serum samples at 4°C overnight. After washing, the bound solFcµR was detected by sequential addition of biotin-labeled mouse anti-FcµR mAb [clone HM14 (γ1κ)] and alkaline phosphatase-labeled streptavidin (AP-SA), before addition of the substrate *p*-nitrophenylphosphate (SIGMA). Unlabeled or biotin-labeled, irrelevant isotype-matched mAbs were used as controls. The enzyme reaction was measured by the absorbance at 405 nm with Spectramax i3x microplate reader (Molecular Devices, San Jose, CA). The results with serum FcµR titers were expressed as an arbitrary unit defined as follows. A given OD value in the linear range of serially diluted serum samples was determined to correspond the OD value of serially diluted standard culture supernatants from solFcµR-His transductants. The reciprocal dilutions of serum samples were divided by the reciprocal dilutions of the corresponded standards, and the resultant value was multiplied by 10 as an arbitrary unit.

For direct ELISA, 96-well hard plates were directly coated with serial dilutions of solFcµR transductant culture supernatants containing 10% FCS proteins, masked with BSB/1% BSA, and then incubated with predetermined concentrations of anti-FcµR or control mAbs before developing with AP-labeled polyvalent goat anti-mouse IgG Abs [Southern Biotechnology Associates (SBA), Birmingham, AL]. In some experiments, Ni-coated plates (Thermo Scientific) or various concentrations of with anti-His mAb (Biolegend), instead of anti-FcµR mAbs, as a developer were used for assessment of His-tagged solFcµR. Frozen serum samples from CLL patients and age-matched healthy controls (six each) were used after obtaining written informed consent before their enrollment in the study. Studies involving human materials were approved by the institutional review boards of Feinstein Institute for Medical Research and Charité Universitätsmedizin.

### Flow Cytometric Analyses of Cell Surface and Intracellular Components

For cell surface staining of stable transductants, an equal mixture of GFP-negative WT cells and GFP-positive transductants was first incubated with Fcγ blocker (clone 2.4G/75) to mask FcγRs, especially on Ag8 cells, and then sequentially incubated with unlabeled or biotin-labeled, FcµR-specific or irrelevant isotype-matched control mAbs at the predetermined concentration for 20 min on ice. After washing with PBS/0.1% BSA/0.1% sodium azide, bound mAbs were detected by respective addition of PE-labeled goat anti-mouse Ig Abs or PE-SA (SBA) as described (5, 13). For intracellular staining, a mixture of Ag8 GFP-negative WT cells and GFP-positive transductants (~10^6^ cells each) was fixed for 10 min with 200 µl of 8-fold diluted paraformaldehyde (PFA) fixing solution (BD Bioscience) on ice, washed, permeabilized for 10 min with 200 µl of methanol on ice, and washed with PBS/0.1% BSA/0.1% sodium azide before sequentially incubating with unlabeled mAbs and then with Alexa647-labeled goat anti-mouse Ig Abs (SBA). In some experiments for transductants, BD Perm/Wash Buffer was used for intracellular staining. Stained cells were examined by Becton Dickinson (BD) LSRFortessa flow cytometer along with FACSDiva software (BD Bioscience), and flow cytometric data were analyzed with Flowjo software (BD) (14). In some experiments, PBMCs isolated from healthy individuals were examined for cell surface and intracellular staining with HMD22 mAb as described (5).

### Production of SolFcµR-Specific mAbs

Instead of purifying recombinant solFcµR protein as an immunogen, we employed an *in vivo* differential immunization strategy based on the findings in sheep that Ag-reactive lymphocytes were selectively recruited into the initially antigenically stimulated lymph nodes (15). Briefly, three 8-wk BALB/c mice were s.c. injected with two different Ag8 transductants expressing memFcµR as tolerogen or solFcµR as immunogen. The left footpad was s.c. injected every 3 days with memFcµR transductant (~4 x 10^6^ cells/injection) from d 0 to 15 during the immunization, and the right footpad was s.c. injected with solFcµR-His transductant (10^7^ cells/injection) from d 3 to 15. For the first injection of both tolerogen and immunogen, cells were emulsified with CFA for injection and for the remainder, irradiated cells were suspended in PBS for injection. On day 16, right popliteal lymph node cells (*i.e.*, immunogen-primed) were fused with an equal number of HAT-sensitive, Ig-nonproducing mouse plasmacytoma line (Ag8.653) as previously described (5, 13). Hybridoma clones producing IgG mAb with selective reactivity with the unique C-terminus of solFcµR, but not with the EC of memFcµR and other potential targets (*i.e.*, His tag and Ag8 cellular components), were selected and subcloned by limiting dilution using IgG-depleted FCS (10%) and rIL-6-containing conditioned medium (3%). The Ig isotypes of mAbs were determined with enzyme-labeled Abs specific for each mouse Ig isotype (SBA). HMD22 mAb (γ1κ) was thus selected in this study and purified from culture supernatants by protein G-coupled Sepharose (Cytivia). All studies involving animals were conducted with approval of the Landesamt für Gesundheit und Soziales (Lageso); permission number H 0126/16.

### Mass Spectrometric Analysis of SolFcµR

For purification of solFcµR, Tween 20 was added to His-tagged solFcµR-containing culture supernatants to the final concentration of 0.05% in order to solubilize exosome-bound solFcµR-His, before affinity chromatography. The affinity columns included: (*i*) 7.5 ml of Sepharose 4B beads coupled with BSA (6.7 mg/ml), (*ii*) 2.5 ml of beads coupled with a cocktail of anti-FcµR EC mAbs (HM7, HM12 and HM14; 15 mg of combined) and (*iii*) 3.5 ml of beads with HMD22 anti-solFcµR mAb (3.2 mg/ml). After sequentially absorbing the culture supernatants, first with BSA-coupled column and then with either of the FcµR-reactive columns, the column was extensively washed with BSB, and the bound materials were eluted by 0.05 M glycine-HCl buffer, pH 2.85, followed by immediate neutralization with 3M Tris. The resultant purified solFcµR was resolved on SDS-10% PAGE under both reducing and non-reducing conditions and stained with Coomassie Brilliant Blue (CBB). The major broad band of ~40 kDa in both reducing and non-reducing gels was excised and subjected to mass spectrometric analysis (10). In brief, the destained gel piece was reduced, alkylated, and then digested with trypsin before extracting peptides. For nano-chip liquid chromatography-tandem mass spectrometry, an aliquot of extracted peptides was applied onto a PharmaFluidics µPAC reverse-phase trapping column (ThermoScientific, Waltham, MA), and the bound peptides were flushed onto a PharmaFluidics 50cm µPAC reverse-phase column 100Å. A SCIEX 5600 Triple-Tof mass spectrometer (Sciex, Toronto, Canada) was used to analyze the protein digest. Eluted peptides were subjected to a time-of-flight survey scan from 400-1250 *m/z* to determine the top twenty most intense ions for MSMS analysis. Product ion time-of-flight scans (50 msec) were carried out to obtain the tandem mass spectra of the selected parent ions over the range from *m/z* 100-1500. Spectra are centroided and de-isotoped by Analyst software, version 1.81 TF (Sciex). A β-galactosidase trypsin digest was used to establish and confirm the mass accuracy of the mass spectrometer. The tandem mass spectrometry data were processed to provide protein identifications using an in-house *Protein Pilot 5.0* search engine (Sciex) using the *Homo sapiens* UniProt protein database and a trypsin plus missed cleavage digestion parameter. Post-translational modifications identified in the software were then verified by manual *de novo* sequencing for authentication.

### Western Blot Analysis

Affinity-purified solFcµR (1 µg) was resolved on SDS-10% PAGE, transferred onto membranes, immunoblotted first with isotype-matched control, solFcµR-specific HMD22 or FcµR EC-specific HM6 mAbs and then with biotin-labeled goat anti-mouse IgG1 Abs, and developed with HRP-SA, before visualization by ECL. For serum FcµR from CLL patients, 50 µl of sera were incubated with 10 µl of Sepharose 4B (50% gel slurry) coupled with HM6, HMD22 or isotype-matched control mAbs (3-4 mg/ml), and the bound materials were dissociated and separated on SDS-10% PAGE, followed by transfer onto membranes. The membranes were sequentially blotted with biotinylated HM14 anti-FcµR mAb and with HRP-SA, before visualization by ECL as described before (10).

### Sequence Analyses of Ig Heavy and Light Chain Variable Regions

Nucleotide sequence of Ig H and L chain V regions of HMD22 mAb was determined by reverse transcription PCR. In brief, the total RNA isolated from the HMD22 hybridoma clone producing a solFcµR-specific mAb of IgG1κ isotype was converted to first-strand cDNA by using SuperScript™ IV First-Strand Synthesis System (Invitrogen) with an oligo(dT)_18_. The resultant first strand cDNA was used as a template DNA for amplification of cDNA encoding Ig H and L chain variable (IGHV and IGKV) regions with a set of primers: (*i*) DO-021 (5’-aggtsmarctgcagsagtcwgg-3’) and DO-023 (5’-ggacagggmtccakagttcc-3’) corresponding with universal VH leader and Cγ for IGHV; and (*ii*) DO-024 (5’-ccagatgtgtgatgacccagactcca-3’) and DO-025 (5’-gttggtgcagcatcagc-3’) corresponding with Vκ leader and Cκ for IGKV (13). (In IUPAC nt code, K = G or T; M = A or C; R = A or G; S = G or C; W = A or T) Each amplification reaction underwent 35 cycles of: denaturation at 94°C for 1 min, annealing at 62°C for IGHV or 54°C for IGKV for 20 sec, and extension at 62°C for 80 sec. A final extension was performed at 72°C for 10 min. The amplified products with the expected size (~540 bp for IGHV and ~350 bp for IGKV) were gel-purified and subcloned into the ZeroBlunt TOPO vector (Invitrogen) before sequencing analysis. The sequence was analyzed by IMGT/V-Quest program (16).

### Statistical Analysis

All data comparisons were performed by nonparametric Mann-Whitney U test (one-tailed), and *P* value of <0.05 was defined as statistically significant.

## Results

### Establishment of Unique Plasmacytoma Lines Stably Producing Recombinant Soluble FcµR Proteins

**Figure 1:**
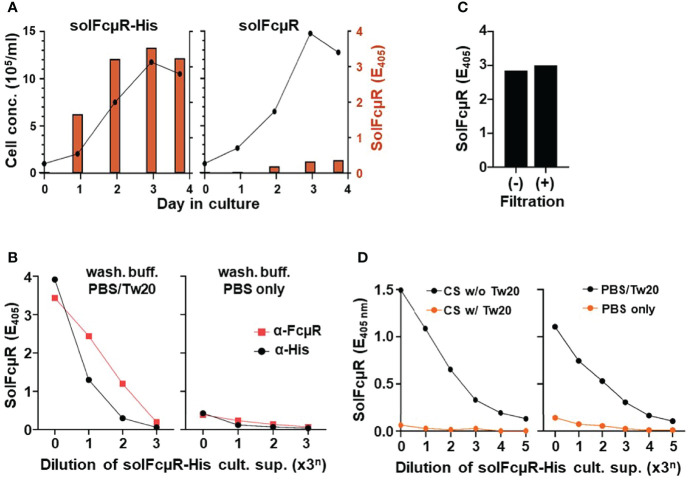
Assessment of solFcµR recombinant proteins in culture supernatants of stable Ag8 transductants by ELISA. **(A)** Comparative analysis of cell growth (line graphs) and solFcµR production (bar graphs) between Ag8 stable transductants of His-tagged (left) and native (right) solFcµR. SolFcµR in culture supernatants was assessed by sandwich ELISA using HM7 anti-FcµR mAb for coating and biotin-labeled, HM14 anti-FcµR mA for developing and a washing buffer of PBS/0.05% Tween 20. **(B)** Detergent-dependent, dose response curve of solFcµR produced by His-tagged solFcµR transductant. ELISA plates were pre-coated with HM7 anti-FcµR (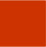) or anti-His (●) mAb, blocked any free sites with BSB/1% BSA, washed with PBS/0.05% Tween 20 (left) or PBS only (right), and then incubated with 3-fold serially diluted culture supernatants of His-tagged solFcµR transductants. After similarly washing, bound solFcµR was detected by addition of biotin-anti-FcµR mAb (HM14), before developing with SA-AP. OD values at 405 nm after 30 min incubation at room temperature with substrate were plotted. **(C)** SolFcµR titers in culture supernatants of His-tagged solFcµR transductant before (–) or after (+) filtration through 0.22 µm filters. **(D)** Right: Polystyrene 96-well plates were coated with 3-fold serially diluted culture supernatants (containing 10% FCS) from His-tagged solFcµR Ag8 transductant, masked with BSB/1% BSA, washed with PBS/0.05% Tween 20 (●) or PBS only (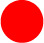), incubated with HM14 anti-FcµR mAb, and developed with AP-goat anti-mouse Ig Ab. OD values were similarly plotted. Left: Plates were similarly coated with culture supernatants containing no (●) or 0.05% Tween 20 (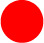), before masking, washing with PBS/0.05% Tween 20, and developing HM14 mAb.

The solFcµR cDNA originally identified in PMA-activated, human pre-B cell line 697, does not contain the transmembrane exon, and hence encodes a unique 70-aa hydrophilic C-terminus due to the reading frame shift (10). We initially transduced this solFcµR cDNA construct into Ag8.653 (hereafter Ag8 in short) plasmacytoma line, but the resultant stable transductant (designated as native solFcµR) produced limited amounts of solFcµR protein (**Figure 1A** right). This assessment was determined by sandwich ELISA using two different FcµR EC-specific mAbs, *i.e.*, its N-terminal Ig-like domain-specific mAbs (*e.g.*, HM6, HM7 or HM10) for capturing, and the stalk region-specific HM14 mAb for developing (10, 13).

We thus modified the native solFcµR cDNA construct. A Kozak sequence “ACC” at the 5’ flanking region of the ATG translation initiation site was introduced to enhance its transcription, and an 18 nt coding six His tag sequence was inserted in front of the TGA translation termination site, before establishing its stable Ag8 transductant (solFcµR-His). The growth of the resultant His-tagged solFcµR transductant was comparable with that of the native solFcµR transductant, but the amount of solFcµR in culture supernatants was ~10-fold higher (**Figure 1A** left). The production of solFcµR by transductants was dependent on cell growth and FCS concentrations in an order: 10 > 3.3 > 1.1% (not shown). Intriguingly, we noted a big difference in assessment of solFcµR in culture supernatants between PBS and PBS/0.05% Tween 20 as a washing buffer. Tween-containing PBS yielded much higher titers of solFcµR-His than PBS alone (**Figure 1B**), suggesting Tween detergent was important in the assessment of His-tagged solFcµR proteins in cell-free culture supernatants. Anti-His, instead of anti-FcµR, mAb-coated plates or Ni-coated plates (not shown) also showed a similar dose response curve (**Figure 1B**), confirming the His attachment to solFcµR. This unexpected detergent-dependency raised a possibility that His-tagged solFcµR might associate with extracellular membrane vesicles or exosomes. The filtration of solFcµR-His transductant supernatants through a 0.22 µm filter did not affect their solFcµR titers (**Figure 1C**), the result being in close agreement with the size of exosomes (~40 to 160 nm) (17). Culture supernatants containing His-tagged solFcµR specifically bound to wells pre-coated with human or mouse IgM in a dose-dependent manner as determined by biotin-labeled HM14 anti-FcµR mAb (not shown).

Another striking finding was immobilization of solFcµR by directly coating ELISA plates with culture supernatants containing His-tagged solFcµR and even 10% FCS proteins (**Figure 1D** left). Anti-FcµR mAbs with either specificity for its Ig-like domain or the stalk region significantly bound to the wells pre-coated with even 81 (3^4^)-fold diluted culture supernatants containing possibly exosome-associated, His-tagged solFcµR (CS w/o Tw20). By contrast, the immobilization of solFcµR was not observed when Tween 20 was added to solFcµR-His-containing culture supernatants (CS w/Tw20), suggesting that once exosome-associated, His-tagged solFcμR was solubilized by addition of detergent, such “solubilized” His-tagged solFcμR could not coat anymore ELISA plates. The essentially same results were also obtained with anti-His mAb, instead of anti-FcµR mAbs, as a developer and with human or mouse IgM paraprotein as a ligand (not shown). The assessment of anti-FcµR mAb binding with the immobilized solFcµR-His was also detergent-dependent; Tween-containing PBS (PBS/Tw20) as washing buffer yielded higher titers of solFcμR than PBS only (**Figure 1D** right). These findings suggested that exosome-associated solFcµR-His could be efficiently immobilized on ELISA plates and that inclusion of Tween detergent in washing buffer might stabilize such attached solFcµR-His during assays. In turn, this unique nature of His-tagged solFcµR could be an advantage when screening hybridoma clones secreting solFcµR-specific mAbs by ELISA using plates coated with solFcµR-His transductant’s culture supernatants, instead of purified solFcµR proteins or use of Ni-coated plates.

Collectively, these findings suggested: (*i*) His-tagged solFcµR transductants produce more solFcµR in culture supernatants than native solFcµR transductants; (*ii*) Assessment of His-tagged solFcµR by ELISA is detergent-dependent, possibly due to association of solFcµR-His with exosomes; and (*iii*) His-tagged solFcµR can be immobilized on ELISA plates by simply coating with transductant culture supernatants containing even 10% FCS proteins.

### Association of His-Tagged, but Not Native, SolFcµR With Plasma Membranes

To explore the molecular basis for association of His-tagged solFcµR with exosomes, we conducted several experiments. *First*, to examine if such association is a unique phenomenon for this particular solFcµR-His transductant, we made another stable Ag8 and an HEK (human embryonic kidney cell line) transductant with the same solFcµR-His cDNA construct. Both Ag8 and HEK stable exhibited the same results after approximately one month after sorting GFP**^hi^** cells, suggesting that such membrane attachment phenomenon was indeed reproducible and might be a slow process.

**Figure 2:**
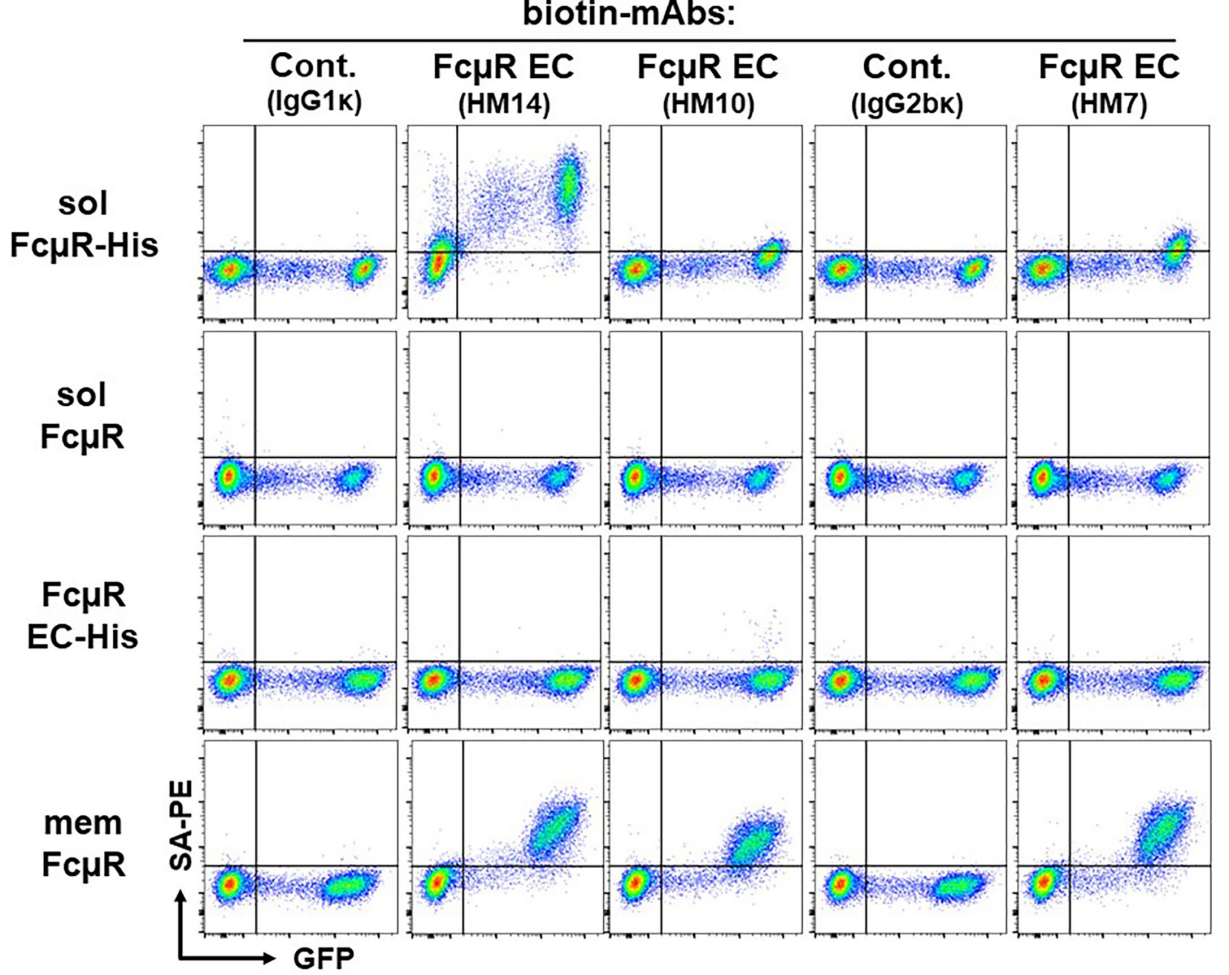
Cell surface flow cytometric analysis of various FcµR transductants. An equal mixture of GFP-negative WT control cells and GFP-positive Ag8 transductants expressing His-tagged solFcµR (solFcµR-His), native solFcµR (solFcµR), His-tagged FcµR EC only (FcµR EC-His) or membrane FcµR (memFcµR), was first incubated with Fcγ blocker and then with the indicated biotinylated mouse mAbs specific for human IgM (SA-DA4 clone, IgG1κ isotype), human FcµR EC (HM14 and HM10 clones, both IgG1κ; HM7 clone, IgG2bκ) or mouse FcµR (MM24, IgG2bκ). The bound biotinylated mAbs were detected by addition of PE-labeled streptavidin (SA-PE). The stained cells were analyzed by LSRFortessa. Note the cell surface staining of His-tagged solFcµR transductant. One of the representative profiles from at least four independent experiments is demonstrated.

*Second*, to determine if His-tagged solFcµR also associates with plasma membranes, we performed a cell-surface flow cytometric analysis. To our surprise, the cell surface of solFcµR-His transductants of both Ag8 (**Figure 2** *top* panel) and HEK (not shown) cells was clearly reactive with both FcµR stalk region-specific HM14 and anti-His mAbs, and weakly, but significantly, with FcµR Ig-like domain-specific mAbs (HM10 and HM7). Irrelevant, isotype-matched (IgG1κ and IgG2bκ) control mAbs did not react with none of the Ag8 transductants as well as with GFP-negative, wild-type (WT) Ag8 cells. These findings are consistent with solFcµR-His attaching to the plasma membrane of its transductant. [The findings that the weak reactivity of HM14, but not isotype-matched control, mAb with GFP-negative cells of the solFcμR-His transductant appeared to be due to insufficient blocking of FcγRs with FcγR blockers. Alternatively, the six His-tag itself might attach to certain membrane components of WT Ag8 cells.] As expected, none of the anti-FcµR mAbs reacted with the cell surface components of Ag8 transductants expressing native solFcµR (*2nd* panel) or His-tagged FcµR EC only (*3rd* panel). Both EC-specific (*i.e.*, Ig-like domain and stalk region) FcµR mAbs equally reacted with the cell surface of transductant expressing membrane form of FcµR (memFcµR) (*bottom* panel). Thus, His-tagged solFcµR seemed to associate with exosomes as well as with plasma membranes, and the epitopes on solFcµR-His proteins recognized by three different mAbs (*i.e.*, HM14, HM10, HM7) did not appear to be equally available, especially in the Ig-like domain. The membrane-attached His-tagged solFcµR might be advantage in generating mAbs, because plasma membrane-bound Ags were shown to be more immunogenic than intracellular Ags.

*Third*, since FcµR is a type I transmembrane protein, and solFcµR lacks the transmembrane exon (5, 10), the solFcµR must be within the membrane vesicles (or luminal phase) in the secretory pathway. How the cell surface of solFcµR-His transductant could be reactive with receptor-specific mAbs without prior cell permeabilization. In this regard, we ruled out the possibility of attachment of solFcµR-containing apoptotic bodies to plasma membranes by lack of staining of solFcµR-His transductants with Annexin V, which specifically recognizes the phosphatidylserine translocated from the inner to the outer leaflet of plasma membranes during apoptosis (18, 19). We also excluded the possibility of GPI-linkage of His-tagged solFcµR by resistance of solFcµR-His transductants to phosphatidylinositol-specific phospholipase C treatment.

Collectively, these findings suggest that certain post-translational modifications, including protein lipidation (or palmitoylation) (20, 21) or uncleaved signal peptide (22–24) of His-tagged solFcµR (see below) or the six His sequence itself, might be responsible for solFcμR membrane attachment.

### Production of HMD22 Hybridoma MAb Specific for Human SolFcµR

In order to develop solFcµR-specific mAbs, we employed an *in vivo* differential immunization strategy by initial s.c. injection of memFcµR Ag8 transductant as a tolerogen at one side of footpad, followed by s.c. injection of solFcµR-His Ag8 transductant as an immunogen at the other side of footpad. This was based on the concept of preferential selection of Ag-reactive lymphocytes into the initially antigenically stimulated lymph nodes (15). Popliteal lymph node cells (4.9 x 10^7^) from the immunogen side of differentially hyper-immunized BALB/c mice were thus fused with an equal number of Ag8 cells and plated into thirteen 24-well plates at ~1.6 x 10^5^ cells/1.5 ml/well. Nearly all (99%) wells contained growing hybridomas, and 49 of the total 312 wells (~16%) contained IgG Abs reactive with solFcµR-His immobilized ELISA plates. Of the 49 ELISA-positive hybridomas, 8 were selected by cell surface flow cytometric analysis based on: (*i*) unreactivity with membrane FcµR expressed by Ag8 and BW5147 thymoma (hereafter BW in short) transductants as well as with their WT control Ag8 and BW cells and (*ii*) reactivity with His-tagged solFcµR attached to plasma membranes of its Ag8 transductant. The final screening was conducted by intracellular flow cytometric analysis using paraformaldehyde (PFA)-fixed and saponin-permeabilized, native solFcµR transductant. One of the 8 hybridomas (clone HMD22) stained the native solFcµR transductant intracellularly, but not WT Ag8 cells, suggesting specificity for the unique C-terminal portion of solFcµR. Thus, we obtained at least one hybridoma candidate secreting a solFcµR-specific mAb from mice differentially hyper-immunized with membrane (tolerogen) and soluble (immunogen) FcµR-expressing cells, along with advantage of unique characteristics of the latter His-tagged solFcµR transductant.

### Specificity of HMD22 MAb to Human Soluble, but Not Membrane, FcµR

After purifying HMD22 mAb from the culture supernatants of its single cell-derived hybridoma clone by using protein G-coupled affinity columns, we further characterized its isotype, variable region nt sequence, and fine specificity. HMD22 was found to be an IgG1κ mAb by ELISA using mouse Ig isotype-specific reagents. The variable regions of heavy and light chains of HMD22 mAb were amplified from the 1st strand cDNA by RT-PCR using a set of universal VH and Cγ and of Vκ and Cκ primers. The nt sequence analysis of the resultant PCR products using the IMGT/V-Quest program (16) revealed that HMD22 mAb utilized IGHV9-4*02 (94.5% identity), IGHD2-14*01, and IGHJ2*01 (93.8%) and IGKV8-24*01 (97.3%) and IGK J5*01 (100.0%) (see **Supplementary Information Figure 1**).

**Figure 3:**
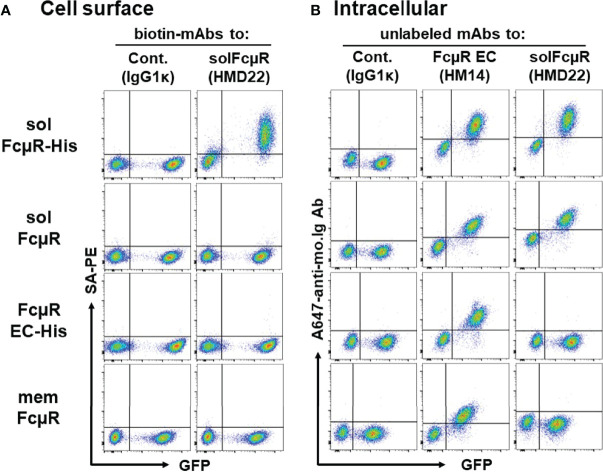
Cell surface and intracellular flow cytometric analyses of transductants with HMD22 mAb. **(A)** An equal mixture of GFP-negative WT control and GFP-positive Ag8 transductants expressing His-tagged solFcµR (solFcµR-His), native solFcµR (solFcµR), His-tagged FcµR EC only (FcµR EC-His) or membrane FcµR was first incubated with Fcγ blocker and then with biotin-labeled, solFcµR-specific mAb (HMD22) or its isotype-matched, irrelevant control mAb (IgG1κ), before developing with SA-PE. **(B)** The same mixture of cells was fixed with 0.5% PFA, washed, and permeabilized with methanol, before intracellular staining with the indicated mouse mAbs. The bound mAbs were detected by addition of Alexa 647-labeled goat anti-mouse Ig Abs. Note that HMD22 mAb intracellularly reacts with both His-tagged and naive solFcµR transductants but not with memFcµR nor His-tagged FcµR EC only transductants. One of the representative results from three independent experiments **(A, B)** is shown.

By flow cytometric analysis, HMD22 mAb did not react with the cell surface components (**Figure 3A**) of Ag8 transductants expressing either of membrane FcµR (*bottom*), naive solFcµR (*2nd*) or His-tagged EC only FcµR (*3rd*) but did react with the cell surface of His-tagged solFcµR Ag8 transductant (*top*). By contrast, when these transductants were fixed with PFA and permeabilized with methanol before intracellular staining with mAbs, HMD22 mAb reacted only with solFcµR transductants, irrespective of presence or absence of His tag; HMD22 mAb did not react with transductants expressing memFcµR or His-tagged EC only FcµR as well as with WT Ag 8 cells (**Figure 3B**). FcµR EC-specific HM14 mAb reacted with all transductants regardless of membrane, soluble or only EC types of FcµR, but not with WT Ag8 cells. Thus, these flow cytometric findings strongly indicate specificity of HMD22 mAb toward the unique C-tail of solFcµR, but not for the receptor EC nor the His tag introduced at the C-terminus of solFcµR. While HMD22 mAb did not react with any cell surface components of freshly prepared blood MNCs, it appeared to react intracellularly with very tiny subpopulations of MNCs; their precise definition will require further analyses.

### Mass Spectrometric Analysis of HMD22 MAb-Reactive Protein of ~40 kDa

**Figure 4:**
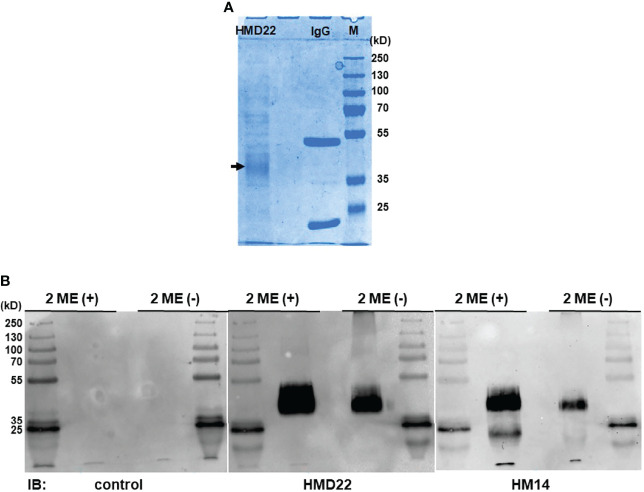
Western blot analysis of HMD22 mAb-reactive proteins. SolFcµR proteins were isolated from culture supernatants containing His-tagged solFcµR and 0.05% Tween 20 by affinity column coupled with a cocktail of EC-specific FcµR mAbs (HM7, HM12 and HM14). The resultant solFcµR proteins of ~15 µg and 1 µg were respectively applied onto SDS-10% PAGE for CBB staining **(A)** and for Western blot analysis **(B)** with immunoblotting with the indicated mAbs: isotype-matched control, solFcµR-specific HMD22, and FcµR EC-specific HM14 mAbs. Note that the major protein with an *M*_r_ of ~40 kDa under both 2-ME (+) and 2-ME (–) is reactive with both HMD22 and HM14 mAbs. Essentially the same results were obtained with solFcµR proteins isolated by affinity column coupled with HMD22 mAb. *M*_r_ markers were PageRuler™ plus pre-stained protein ladder 10 to 250 kDa (Thermo Scientific, Cat. No. 26620).

To confirm further the specificity of HMD22 mAb and to explore any potential post-translational modification(s) responsible for membrane attachment of His-tagged solFcµR, we purified solFcµR from the culture supernatants containing solFcµR-His and 0.05% Tween 20 using affinity columns coupled with either a cocktail of FcµR EC-specific mAbs or solFcµR-specific HMD22 mAb for mass spectrometric analyses. Almost comparable amounts of solFcµR protein were recovered from 800 - 900 ml of culture supernatants (~0.3 mg for the former and ~0.4 mg for the latter). Both solFcµR-His preparations were resolved as a major broad protein band with an *M*_r_ of ~40 kDa on SDS-10% PAGE under both reducing and non-reducing conditions (**Figure 4A**). Several minor proteins with higher *M*_r_ were also detected, especially under reducing conditions, and might be irrelevant proteins associated with the micelles containing solFcµR-His. By Western blot analysis, both HMD22 and HM14 mAbs reacted with the major broad band of ~40 kDa, and this occurred more strongly under reducing than non-reducing conditions suggesting the possibilities of variable degrees of O-glycosylation and of the covalent association of a portion of solFcµR-His protein with other larger proteins (**Figure 4B**). An additional smaller band of ~15 kDa was also detected under reducing conditions by immunoblotting with HM14, but not HMD22, mAbs, suggesting that the cleaved solFcµR-His lacking an HMD22-reactive epitope might covalently associate with other proteins.

**Figure 5:**
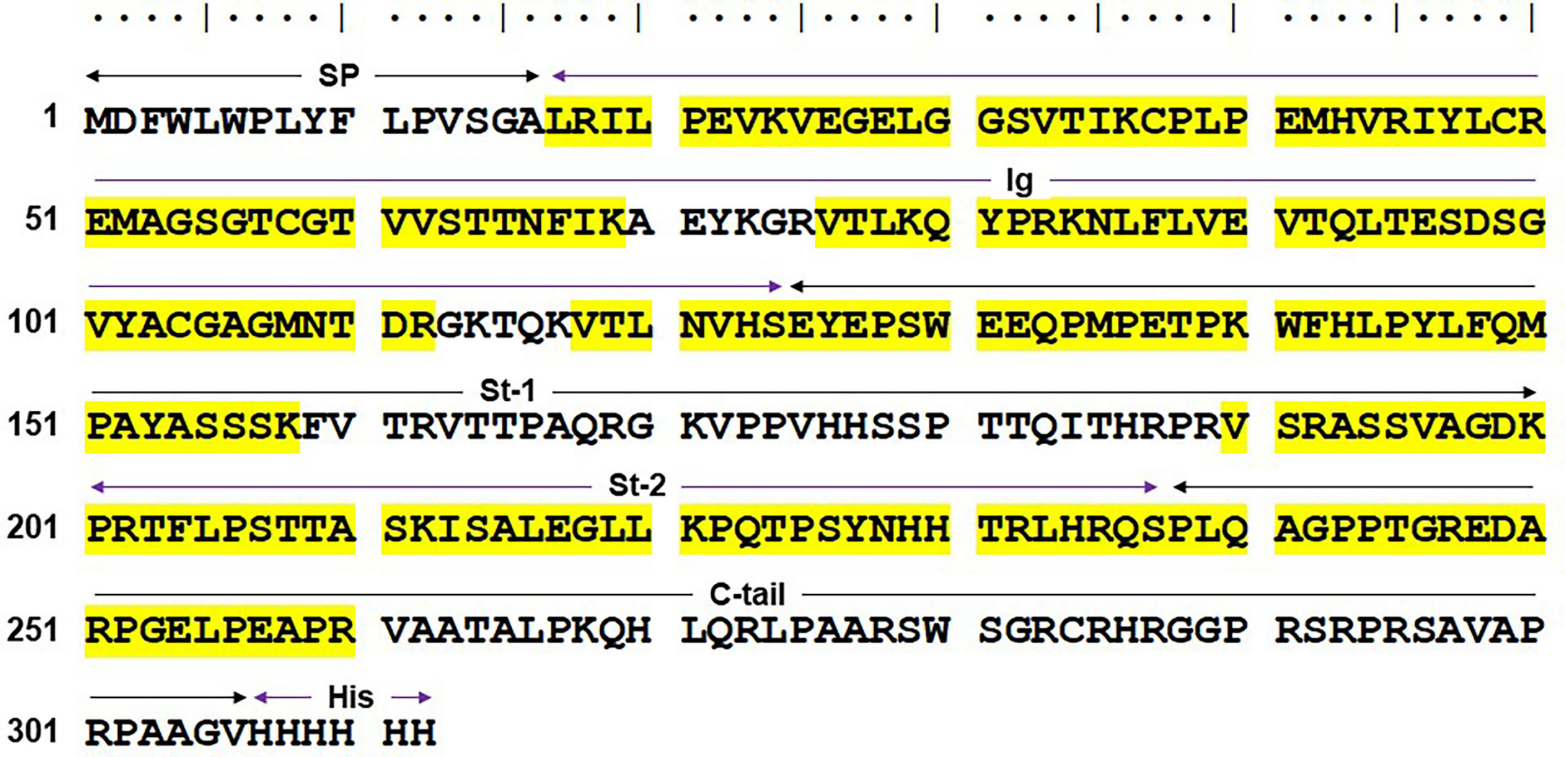
Mass spectrometric results of the HMD22 mAb-reactive, ~40 kDa protein. The major ~40 kDa band of HMD22 mAb-reactive solFcµR-His proteins purified from culture supernatants was subjected to mass spectrometric analysis. Amino acid sequence (single letter code) deduced from the His-tagged solFcµR is shown along with the sequences identified by the mass spectrometric analysis (highlighted in yellow). Predicted signal peptide (SP)-containing region, Ig-like domain (Ig), stalk regions 1 and 2 (St-1, St-2) common to both membrane and soluble forms of FcµR, the C-terminal tail unique for solFcµR (C-tail), and six His tag (His) are indicated. The sequence identified by mass spectrometric analysis of the tryptic peptides of the ~40 kDa solFcµR-His are highlighted in yellow. The results are demonstrated from the mass spectrometric analyses of two different preparations of HMD22 mAb-reactive protein of ~40 kDa.

Based on these findings, protein bands of ~40 kDa in both reducing and non-reducing conditions were excised from SDS-10% PAGE and subjected to proteomic analysis. The results from liquid chromatography-tandem mass spectrometry analysis revealed that several tryptic peptides in the ~40kDa bands corresponded with the human solFcµR (NCBI ADK11426; UniProtKB-O60667-3), and the matched peptides distributed from the Ig-like domain and stalk to the C-terminal portion of the solFcµR molecule (**Figure 5**). Given the finding that the most N-terminal residue identified was Leu at position of 17, which is predicted to be a signal peptidase cleavage site, the possibility that the uncleaved signal peptide of solFcµR-His was responsible for membrane attachment seemed to be less likely. Alternatively, fatty acid attachment to Ser281 or Cys284 of solFcµR-His induced by introduction of six His residues at the C-terminus was considered based on the high homology between the palmitoylated CD4 at Cys419 and Cys422 sites (**^419^** CVRCRHR**^425^**) (17) and the solFcµR (**^281^** SGRCRHR**^287^**). However, no tryptic peptides covering the C-terminal 52-aa region of solFcµR-His were observed, probably due to the presence of 9 Arg and one Lys residues in the C-terminus or the inability of trypsin to access this potentially membrane-attached region.

Several other post-translational modifications of solFcµR-His were identified: (*i*) phosphorylation of Ser129; (*ii*) methylation of: Glu at positions of 28, 41*, 127*, 131*, 254 and 257^§^, Lys36^§^, and Arg at positions of 45^§^, 247^§^, 251^§^ and 260^§^; (*iii*) deamidation of: Gln at positions of 149 and 240^§^, and Asn166; (*iv*) deimidation or citrullination of Arg at positions of 192 and 235; (*v*) trioxidation of Cys37; (*vi*) dioxidation of Met at positions of 41 and 150^§^; (*vii*) oxidation of Pro at positions of 38* and 252; and (*viii*) glutathionylation of Cys37*. (* and ^§^ indicate modifications found in the only band from non-reducing and reducing gel, respectively.) Determining the significance of these post-translational modifications in receptor function will require further experiments. Collectively, the results from proteomic analysis confirm the specificity of HMD22 mAb for solFcµR and define several post-translational modifications.

### Assessment of Serum FcµR in CLL Patients and Age-Matched Healthy Individuals by ELISA

**Figure 6:**
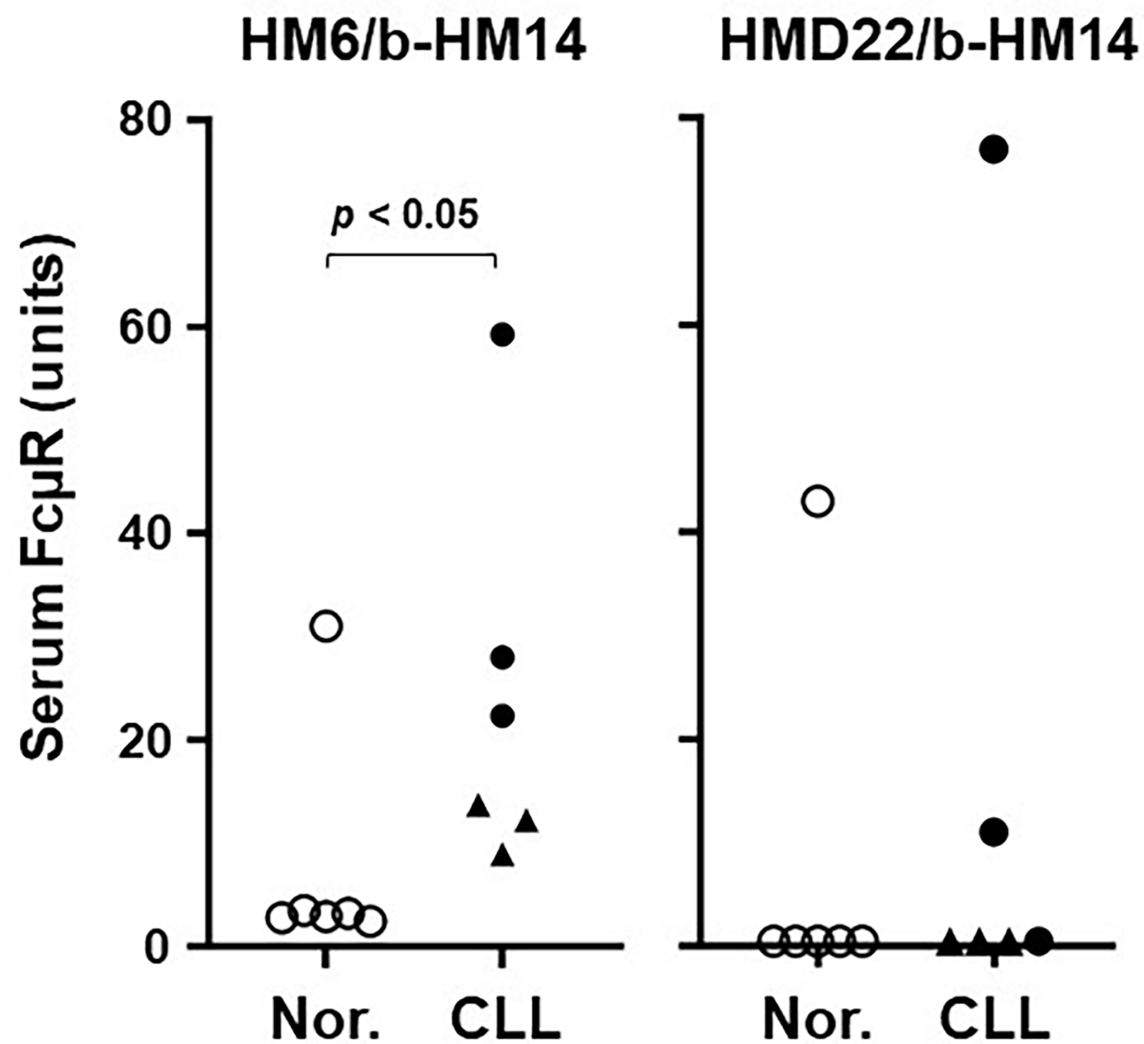
Serum levels of FcµR as determined by sandwich ELISA. Wells pre-coated with FcµR EC-specific HM6 (left) or solFcµR-specific HMD22 (right) mAbs were incubated with serial dilutions of serum samples from normal donors (○) and *IGHV* unmutated (●) or mutated (▲) CLL patients. The bound FcµR was detected by addition of biotin-labeled HM14 anti-FcµR mAb (b-HM14), before developing by addition of AP-SA. Serum levels of FcµR are expressed as arbitrary units of FcµR as described in Materials and Methods. Data comparison was performed by nonparametric Mann-Whitney U test (one tailed) and *P* value of < 0.05 was defined as statistically significant.

In our previous assessments of solFcµR in CLL patients’ sera, two different EC-specific FcµR mAbs were used in sandwich ELISA (HM6 for capturing and HM14 for developing). Unfortunately, the assay with this combination might not discriminate between splice variant (*i.e.*, soluble form of FcµR with an *M*_r_ of ~40 kDa) and cleaved membrane FcµR (*i.e.*, shedding FcµR with an *M*_r_ of ≤~35 kDa), unless the *M*_r_ estimation of serum FcµR was employed for every sample. Thus, we compared the serum titers of FcµR between sandwich ELISA using HM6 and HMD22 mAb as a capturing mAb among a limited number of CLL and age-matched healthy donors. In the EC-specific HM6 mAb assay, all 6 CLL serum samples [3 *IGHV*-unmutated (U-CLL) and 3 *IGHV*-mutated (M-CLL)] contained significantly higher titers of FcµR, with a trend for higher levels in U-CLL than M-CLL, as compared with each age-matched healthy control except one (**Figure 6**), consistent with the previous results (10). Intriguingly, one apparently healthy individual, who is a sister of a CLL patient, had also a high titer of serum FcµR. By contrast, in the solFcµR-specific HMD22 mAb assay, only 2 U-CLL serum samples, the top two in the HM6 assay system, as well as the normal outlier had detectable FcµR. Subsequent pilot analysis of serum FcμR from each CLL patient with the double [HM6(+)/HMD22(+)] or single [HM6(+)/HMD22 (–)] positive phenotype revealed that HM6 mAb clearly precipitated a protein with an M*_r_* of ~40 kDa from both double and single positive CLL patients, whereas HMD22 anti-solFcμR mAb did not bring down clear bands except for non-specific background bands of IgG heavy and light chains at an optimal exposure time point. Upon longer exposure to ECL reagents, however, a very faint and fuzzy band of similar M*_r_* of ~40 kDa was observed in the HMD22-precipitated lane from the double, but not single, positive CLL patient (see **Supplementary Figure 2**). These findings thus suggested that the lack of HMD22-reactivity with serum FcμR in subpopulations of CLL patients did not result from shedding of membrane-bound FcμR, but rather from its lower affinity for serum solFcμR compared to HM6 mAb. This conclusion was consistent with the findings that the HMD22-reactivity with serum samples was correlated with high titers of serum FcμR assessed by HM6 mAb. Alternatively, HMD22 mAb might more preferentially recognize a ligand-bound form of solFcµR than a free form of solFcµR. This possibility was deduced by the findings that the reactivities of HM14 and HMD22 mAbs with cell surface of solFcµR-His transductant were much stronger than those of HM7 and HM10 mAbs, which recognize an epitope near the ligand binding site (see **Figures 2**, **3A**).

Collectively, these findings suggest the importance of both sandwich ELISAs using EC-specific HM6 and solFcµR-specific HMD22 mAbs to capture serum FcµR in CLL.

## Discussion

The aim of present study was to establish solFcµR-specific sandwich ELISA, so that we will be able to test the hypothesis that the production of solFcµR is the consequence of chronic stimulation of B cells *via* the BCR and thus, the serum levels of solFcµR in individuals with diseases characterized by persistent BCR signaling might be elevated. By taking advantages of unique characteristics of Ag8 transductant stably expressing His-tagged solFcµR recombinant protein and an *in vivo* differential immunization strategy, we successfully developed a mouse IgG1κ hybridoma mAb specific for human solFcµR (HMD22 clone).

*Unique feature of His-tagged solFcµR transductant.* Initially we attempted to isolate solFcµR from the culture supernatants of Ag8 cells stably transduced by native solFcµR cDNA. However, because of poor production, we modified the native solFcµR cDNA construct, introducing an ideal Kozak sequence at 5’ flanking of the translation initiation site and inserting an 18 nt coding six His residues before the termination codon. The resultant His-tagged solFcµR transductant produced > 10-fold more solFcµR compared with the native solFcµR transductant as determined by sandwich ELISA. To our surprise, unlike native solFcµR, His-tagged solFcµR protein associated with membranes rather than being in a free, secreted form. Several findings supported this notion. (*i*) ELISA assessment of His-tagged solFcµR in culture supernatants was greatly improved by inclusion of Tween 20 detergent in washing buffer, and solFcµR titers were not affected by filtration of the supernatants through 0.22 µm filters. This suggested attachment to exosomes. (*ii*) The His-tagged solFcµR transductant supernatants containing even 10% FCS could be used for directly coating ELISA plates to immobilize solFcµR-His, implying that exosome-associated proteins might efficiently adhere to polystyrene surface than their soluble free forms. This unique property provided the initial convenient screening strategy by ELISA for hybridomas secreting solFcµR-specific mAbs without purifying solFcµR proteins. (*iii*) His-tagged solFcµR also attached to plasma membrane as determined by cell surface immunofluorescence analysis using receptor-specific mAbs. This feature could be an advantage in raising mAbs against solFcµR, because protein Ags attached to plasma membranes are thought more immunogenic than intracellular protein Ags. (*iv*) SolFcµR has a unique motif ^281^SerGlyArgCysArgHisArg^287^ near the C-terminus, homologous to the sequence of ^419^CysValArgCysArg-HisArg^425^ in the cytoplasmic tail of palmitoylated CD4 at Cys419 and Cys422 (20). Thus, it is conceivable that either Ser^281^ or Cys^284^ in His-tagged solFcµR, unlike native solFcµR, may receive a similar palmitoylation modification, thereby accounting for its membrane attachment. Unfortunately, the present proteomic analysis did not verify this possibility, because tryptic fragments covering the 52-aa C-terminus from Val^261^ to His^312^ of His-tagged solFcµR were not found, probably due to inability of trypsin to access such membrane-attached region. (*v*) Alternatively, the membrane association of solFcµR-His might be restricted only to the six His residues’ part. (*vi*) It is unlikely that uncleaved signal peptide of His-tagged solFcµR explain its membrane attachment because the most N-terminal residue of His-tagged solFcµR identified by proteomic analysis was Leu at position 17, where signal peptidase was predicted to cleave during the *de novo* synthesis of solFcµR-His.

*In vivo differential immunization strategy*. Our *in vivo* differential immunization strategy using stable transductants as immunogens and WT cells as tolerogens allowed us to successfully generate a panel of mAbs specific for several cell surface receptors. These included mAbs specific for: human Fcα/µR in mice (Naoto Nishizaki and HK, unpublished), mouse Fcα/µR in rat (Rebekah Wharton and HK, unpublished), human FcµR in mice (5, 13), and mouse FcµR in *Fcmr-*ablated mice (25). This strategy is based on experimental data in sheep that there is a specific selection of Ag-reactive cells from the recirculating lymphocyte pool into primarily Ag-stimulated lymph nodes (15). Since we have never simultaneously compared the incidence of Ag-specific hybridomas in regional lymph node cells between immunogen- and tolerogen-injected sites, we could not formally evaluate how efficient this *in vivo* differentiation immunization was. However, given our previous experimental results and in the present cell fusion yielding at least one solFcµR-specific mAb (HMD22) with specificity for its 70-aa unique hydrophilic C-terminus, the approach seems quite efficient.

*Assessment of serum FcµR by EC-specific HM6 vs solFcµR-specific HMD22 mAbs.* In our previous studies, CLL B cells expressed much higher levels of FcµR on their cell surface than B cells from healthy controls, and this enhancement was more evident in better outcome IGHV-mutated, CD38**^-^**, or early Rai-stage CLL patients than in IGHV-unmutated, CD38**^+^** or advanced Rai-stage patients (10). Furthermore, when assessing serum FcµR levels by sandwich ELISA using the HM6 EC-specific mAb for capturing in 102 CLL patients (22 M-CLL, 25 U-CLL, 55 IGHV untyped) and 31 healthy donors, serum titers of FcµR varied greatly among the CLL patient samples, although many were clearly elevated compared with normal serum samples. Unlike the cell surface FcµR levels, the serum FcµR levels did not correlate with *IGHV* mutation status or Rai stages, but rather with blood lymphocyte numbers (10). Present quantitation of serum FcµR in a limited number of samples with the same HM6 mAb also yielded a clear discrimination between CLL patients and normal donors as before, although serum titers of FcµR appeared to be higher in the U-CLL than the M-CLL group. On the other hand, when the solFcµR-specific HMD22 mAb was employed to capture serum FcµR, only 2 U-CLL samples (the top two in the HM6 mAb-capturing assay) contained detectable amounts of solFcµR, and the remaining 4 sera did not. For this difference in HM6 and HMD22 assays we initially considered four possibilities: (*i*) the cleaved membrane FcμR (shedding) *vs* alternatively spliced FcμR (soluble); (*ii*) the intact *vs* cleaved form of solFcμR; (*iii*) the affinity of HM6 *vs* HMD22 mAb for serum FcμR; or (*iv*) the ligand-bound *vs* free forms of solFcµR. Subsequent analysis of each HM6/HMD22-double positive and only HM6-positive CLL serum revealed that the lack of HMD22-reactivity in certain sera resulted from neither the shedding of membrane FcμR nor the cleavage of solFcμR, but rather from the difference in affinities of HM6 and HMD22 mAbs for serum solFcμR or from preferential recognition of its ligand-bound form by HMD22 mAb.

Intriguingly, apart from CLL samples, one of the six healthy donors in the present study had high serum titers of FcµR determined by both HM6 and HMD22 mAb assays. Notably, this donor was the sister of a CLL patient. Like one exceptional “normal” donor observed in our previous studies (2, 10), this first-degree relative “healthy” donor might provide a clue on the genesis of solFcµR.

*Potential significance of solFcµR.* Various mechanisms are responsible for the generation of soluble receptors, such as cleavage of membrane-bound or GPI-anchored receptors, alternative splicing, and exosome-like vesicles. Many human genes encoding cell surface receptors express multiple transcripts through alternative splicing. Enhanced expression of both membrane-bound and soluble forms of FcµR has been described in CLL (10). Ag-independent, autonomous homotypic BCR-interactions is a hallmark feature of the neoplastic B cells in CLL (26, 27). This interaction dictates the clinical course of disease, with stronger affinities and longer survival in indolent, M-CLL cases *vs* weaker and shorter contacts in aggressive, U-CLL cases (27). Given these findings, we hypothesize that the production of solFcµR is the consequence of chronic BCR stimulation. Thus, the serum levels of solFcµR might be elevated in individuals with other disease characterized by chronic BCR stimulation, such Ab-mediated autoimmune disorders as suggested by one exceptional “normal” donor in our previous study (2, 10). SolFcµR may modulate B cell function, either as a decoy receptor or by interacting with IgM BCR. In this regard, it is noteworthy that administration of another form of recombinant solFcµR (human FcµR EC/IgG Fc fusion protein) into experimental allergic encephalomyelitis-susceptible mice ameliorates the disease (28). Using the newly developed, solFcµR-specific HMD22 mAb, along with the EC-specific HM6 mAb, in sandwich ELISA will allow to conduct rapid, accurate, and large scale monitoring of solFcµR in such patients and to determine its correlation to disease severity and response to treatment as well as providing insight into the generation of solFcµR.

## Data Availability Statement

The datasets presented in this study can be found in online repositories. The names of the repository/repositories and accession number(s) can be found below: https://www.ncbi.nlm.nih.gov/, OM272991 https://www.ncbi.nlm.nih.gov/, OM272992.

## Ethics Statement

The studies involving human participants were reviewed and approved by Institutional review boards of Feinstein Institute for Medical Research and Charité Universitätsmedizin Berlin. The patients/participants provided their written informed consent to participate in this study. The animal study was reviewed and approved by Landesamt für Gesundheit und Soziales (LaGeSo).

## Author Contributions

HK and PMA designed experiments and made stable transductants. HK, PMA and PKJ developed hybridomas. PMA conducted ELISA (**Figures 1**, **6**), flow cytometry (**Figures 2**, **4**) and determined Ig HC and LC V regions of HMD22 hybridoma (**Supplementary Figure 1**). PMA, RT, and HK purified and characterized solFcµR proteins (**Figure 3**). PMA conducted a pilot protein blot analysis (**Supplementary Figure 2**). LW and SB performed proteomic analysis (**Figure 5**). NC and PE provided serum samples. FM and AR discussed and intellectually contributed. HK, NC, AR and FM wrote the paper. All authors reviewed the article and approved its final form.

## Funding

This work was supported by the DRFZ Institutional Research Fund (to AR, FM, HK), S10 RR027822-01 (to SB), DFG TRR130-P16, DFG TRR241-B03, DFG-Projektnummer 389687267, and ERC 2010-AdG.2010317 Grant 268978 (to AR).

## Conflict of Interest

The authors declare that the research was conducted in the absence of any commercial or financial relationships that could be construed as a potential conflict of interest.

## Publisher’s Note

All claims expressed in this article are solely those of the authors and do not necessarily represent those of their affiliated organizations, or those of the publisher, the editors and the reviewers. Any product that may be evaluated in this article, or claim that may be made by its manufacturer, is not guaranteed or endorsed by the publisher.

## Supplementary Material

Supplementary Figure 1 Nucleotide and translated amino acid sequences of VH and Vκ regions of HMD22 mAb. Both nucleotide and translated amino acid sequences of IGHV-Cγ1 **(A)** and IGKV **(B)** of HMD22 mAb are indicated along with the predicted framework (FR), complementary determining (CDR), diversity **(D)**, joining (JH and Jκ), and constant (Cγ1 and Cκ) regions. The nucleotides highlighted in grey indicate the sites corresponding to primers DO-021 and DO-023 **(A)** and DO-024 and DO-025 **(B)**. Sequence analysis with IMGT/V-Quest program reveals IGH V9-4*02 (94.5%), D2-14*01 and J2’01 (93.8%) and IGK V8-24*01 (97.3%) and J5*01 (100%). Gene accession numbers of NCBI for Ig HC and LC variable regions of HMD22 mAb are OM272991 and OM272992, respectively.

Supplementary Figure 2 Protein blot analysis of serum FcμR in CLL patients with the phenotype of HM6(+)/HMD22(+) versus HM6(+)/HMD22(-). Fifty μl of CLL patient sera with the phenotype of either double positive [HM6(+)/HMD22(+)] (lanes 1 – 3) or single positive [HM6(+)/HMD22(-)] (lanes 4 -6) were incubated with 10 μl of 50% slurry of Sepharose 4B beads coupled with isotype-matched control (lanes 1 & 6), HM6 (lanes 2 & 4) or HMD22 (lanes 3 & 5) mAbs (3-4 mg/ml). After extensive washing, bound materials were eluted by 0.5 M Glycine-HCl buffer, pH 2.85, immediately neutralized with Tris, and resolved on SDS-10% PAGE under reducing conditions, followed by transfer onto membranes. The membranes were sequentially blotted with biotin-labeled HM14 anti-FcμR mAb and with HRP-SA before visualization by ECL.

## Abbreviations

Ag8, Ag8.653 Q8 plasmacytoma line; AP-SA, alkaline phosphatase-labeled streptavidin; BSB, borate saline buffer; BW, BW5147thymoma line; CBB, Coomassie Brilliant Blue; CLL, chronic lymphocytic leukemia; FcµR, Fc receptor for IgM; HEK, human embryonic kidney cell line.
