# Supplementary material for: Soluble Fc Receptor for IgM in Sera From Subsets of Patients With Chronic Lymphocytic Leukemia as Determined by a New Mouse Monoclonal Antibody
Source: Front Immunol. 2022 Jun 16;13:863895. doi: 10.3389/fimmu.2022.863895 (PMC9245419; doi:10.3389/fimmu.2022.863895)
Supplement: Supplementary Figure 1 — Nucleotide and translated amino acid sequences of VH and Vκ regions of HMD22 mAb. Both nucleotide and translated amino acid sequences of IGHV-Cγ1 (A) and IGKV (B) of HMD22 mAb are indicated along with the predicted framework (FR), complementary determining (CDR), diversity (D), joining (JH and Jκ), and constant (Cγ1 and Cκ) regions. The nucleotides highlighted in grey indicate the sites corresponding to primers DO-021 and DO-023 (A) and DO-024 and DO-025 (B). Sequence analysis with IMGT/V-Quest program reveals IGH V9-4*02 (94.5%), D2-14*01 and J2’01 (93.8%) and IGK V8-24*01 (97.3%) and J5*01 (100%). Gene accession numbers of NCBI for Ig HC and LC variable regions of HMD22 mAb are OM272991 and OM272992, respectively. [file DataSheet_1.pdf]

HMD22 mAb IGHV-Cγ1

Fig. S1A

FR1

a ggt cca gct gca gga gtc tgg atc tcc tgc aag gct tct ggg tat 46
1 Gly Pro Ala Ala Gly Val Trp Ile Ser Cys Lys Ala Ser Gly Tyr 15

CDR1 FR2

47 acc ttc aca act gct gga atg cac tgg gtg caa aag atg cca gga 91
16 Thr Phe Thr Thr Ala Gly Met His Trp Val Gln Lys Met Pro Gly 30

CDR2

92 aag gct ttg aag tgg att ggc tgg ata aac acc cac tct gga gtg 136
31 Lys Ala Leu Lys Trp Ile Gly Trp Ile Asn Thr His Ser Gly Val 45

137 cca aaa tat gca gaa gac ttc aag gga cgg ttt gcc ttc tct ttg 181
46 Pro Lys Tyr Ala Glu Asp Phe Lys Gly Arg Phe Ala Phe Ser Leu 60

FR3

182 gaa acc tct gcc agc act gca tat tta cag ata agc aac ctc aaa 226
61 Glu Thr Ser Ala Ser Thr Ala Tyr Leu Gln Ile Ser Asn Leu Lys 75

D

227 aat gag gac acg gct acg tat ttc tgt gcg gta gag ggc ttt gac 271
76 Asn Glu Asp Thr Ala Thr Tyr Phe Cys Ala Val Glu Gly Phe Asp 90

JH

272 tac tgg ggc caa ggc acc act ctc aca gtc tcc tca gcc aaa acg 316
91 Tyr Trp Gly Gln Gly Thr Thr Leu Thr Val Ser Ser Ala Lys *Thr* 105

Cγ1

317 aca ccc cca tct gtc tat cca ctg gcc cct gga tct gct gcc caa 361
106 Thr Pro Pro Ser Val Tyr Pro Leu Ala Pro Gly Ser Ala Ala Gln 120

362 act aac tcc atg gtg acc ctg gga tgc ctg gtc aag ggc tat ttc 406
121 Thr Asn Ser Met Val Thr Leu Gly Cys Leu Val Lys Gly Tyr Phe 135

407 cct gag cca gtg aca gtg acc tgg aac tct gga tcc ctg tcc 451
136 Pro Glu Pro Val Thr Val Thr Trp Asn Ser Gly Ser Leu Ser 149

HMD22 mAb IGKV-Cκ

```
                                                              FR1
1    cc aga tgt gtg atg acc cag act cca tcc tcc ctg gct atg tca gta    47
1       Arg Cys Val Met Thr Gln Thr Pro Ser Ser Leu Ala Met Ser Val    15

48      gga cag aag gtc act atg agc tgc aag tcc agt cag agc ctt tta    92
16      Gly Gln Lys Val Thr Met Ser Cys Lys Ser Ser Gln Ser Leu Leu    30

              CDR1
93      aat agt agc aat caa aag aac tat ttg gcc tgg tac cag cag aaa   137
31      Asn Ser Ser Asn Gln Lys Asn Tyr Leu Ala Trp Tyr Gln Gln Lys    45

          FR2                                     CDR2
138     cca gga cag tct cct aaa ctt ctg ata tac ttt gca tcc act agg   182
46      Pro Gly Gln Ser Pro Lys Leu Leu Ile Tyr Phe Ala Ser Thr Arg    60

183     gaa tct ggg gtc cct gat cgc ttc ata ggc agt gga tct ggg aca   227
61      Glu Ser Gly Val Pro Asp Arg Phe Ile Gly Ser Gly Ser Gly Thr    75

          FR3
228     gat ttc act ctt acc atc agc agt gtg cag gct gaa gac ctg gca   272
76      Asp Phe Thr Leu Thr Ile Ser Ser Val Gln Ala Glu Asp Leu Ala    90

                                     CDR3
273     gat tac ttc tgt cag caa cat tat agc act ccg ctc acg ttc ggt   317
91      Asp Tyr Phe Cys Gln Gln His Tyr Ser Thr Pro Leu Thr Phe Gly   105

                Jκ                                  Cκ
318     gct ggg acc aag ctg gag ctg aaa cgg gct gat gct gca cca ac    362
106     Ala Gly Thr Lys Leu Glu Leu Lys Arg Ala Asp Ala Ala Pro       120
```
