# Supplementary material for: Soluble Fc Receptor for IgM in Sera From Subsets of Patients With Chronic Lymphocytic Leukemia as Determined by a New Mouse Monoclonal Antibody
Source: Front Immunol. 2022 Jun 16;13:863895. doi: 10.3389/fimmu.2022.863895 (PMC9245419; doi:10.3389/fimmu.2022.863895)
Supplement: Supplementary Figure 2 — Protein blot analysis of serum FcμR in CLL patients with the phenotype of HM6(+)/HMD22(+) versus HM6(+)/HMD22(-). Fifty μl of CLL patient sera with the phenotype of either double positive [HM6(+)/HMD22(+)] (lanes 1 – 3) or single positive [HM6(+)/HMD22(-)] (lanes 4 -6) were incubated with 10 μl of 50% slurry of Sepharose 4B beads coupled with isotype-matched control (lanes 1 & 6), HM6 (lanes 2 & 4) or HMD22 (lanes 3 & 5) mAbs (3-4 mg/ml). After extensive washing, bound materials were eluted by 0.5 M Glycine-HCl buffer, pH 2.85, immediately neutralized with Tris, and resolved on SDS-10% PAGE under reducing conditions, followed by transfer onto membranes. The membranes were sequentially blotted with biotin-labeled HM14 anti-FcμR mAb and with HRP-SA before visualization by ECL. [file DataSheet_2.pdf]

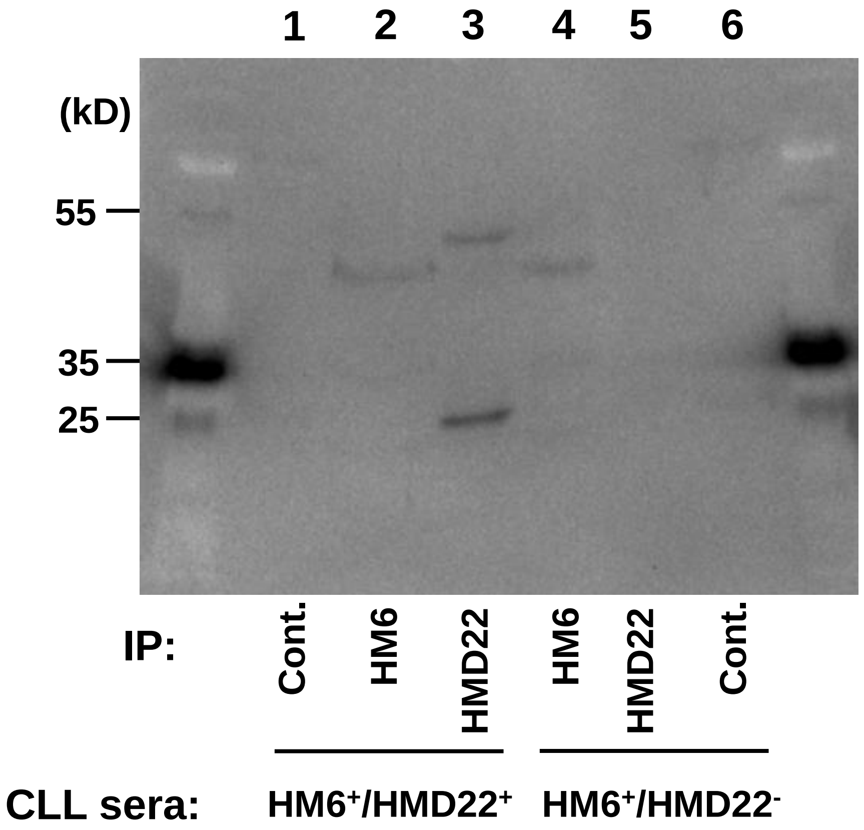

Fig. S2. Protein blot analysis of serum FcμR in CLL patients with the phenotype of HM6(+)/HMD22(+) versus HM6(+)/HMD22(-). Fifty μl of CLL patient sera with the phenotype of either double positive [HM6(+)/HMD22(+)] (lanes 1 – 3) or single positive [HM6(+)/HMD22(-)] (lanes 4 -6) were incubated with 10 μl of 50% slurry of Sepharose 4B beads coupled with isotype-matched control (lanes 1 & 6), HM6 (lanes 2 & 4) or HMD22 (lanes 3 & 5) mAbs (3-4 mg/ml). After extensive washing, bound materials were eluted by 0.5 M Glycine-HCl buffer, pH 2.85, immediately neutralized with Tris, and resolved on SDS-10% PAGE under reducing conditions, followed by transfer onto membranes. The membranes were sequentially blotted with biotin-labeled HM14 anti-FcμR mAb and with HRP-SA before visualization by ECL.
